# Mitigation of acrylamide by l-asparaginase from *Bacillus subtilis* KDPS1 and analysis of degradation products by HPLC and HPTLC

**DOI:** 10.1186/s40064-016-2159-8

**Published:** 2016-04-26

**Authors:** Gaurav Sanghvi, Kapil Bhimani, Devendra Vaishnav, Tejas Oza, Gaurav Dave, Prashant Kunjadia, Navin Sheth

**Affiliations:** Department of Pharmaceutical Sciences, Saurashtra University, Rajkot, 360005 India; Department of Biochemistry, Saurashtra University, Rajkot, India; B. N. Patel Institute of Paramedical Sciences, Bhalej Road, Anand, India; Max Planck Institute of Developmental Biology, Tubingen, Germany

**Keywords:** Acrylamide, HPLC, HPTLC, l-Asparaginase, Solid state fermentation

## Abstract

The use of bacterial l-asparaginase (LA) is one of the alternative approaches for acrylamide reduction in food stuffs as it catalyzes the conversion of l-asparagine to l-aspartic acid and ammonia. In present investigation, purification of extracellular LA from isolate of *Bacillus subtilis* sp. strain KDPS-1 was carried out by solid state fermentation process. The effects of solid substrates, initial moisture content, moistening agents, temperature, and incubation time on LA production was studied, and the highest asparaginase activity (47 IU/ml) was achieved in the medium having orange peel as substrate. The enzyme was purified to homogeneity by diethylaminoethyl (DEAE) cellulose ion exchange chromatography; with 84.89 % yield and 12.11 fold purity. LA showed stimulant activity against β-mercaptoethanol and was greatly inhibited by Zn^2+^ and Hg^2+^ metal ions. Reduction of acrylamide in fried potatoes was detected by high performance liquid chromatography, which showed clear degradation of acrylamide by height and area (%) in the chromatograms of standard sample to that of the test sample. Hydrolysates analysis by high performance thin layer chromatography confirmed the test sample to be LA.

## Background

LA (l-asparagine amido hydrolase, E.C. 3.5.1.1.) is a tetrameric enzyme that catalyzes the hydrolysis of non-essential amino-acid l-asparagine to l-aspartic acid and ammonia (Ruyssen and Lauwers 1978). Free asparagine was found to be the main source of acrylamide which was converted in presence of reducing carbohydrates at temperature above 120 °C (Mottram et al. 2002). It has been confirmed that a wide range of industrial, catering or homemade foods contains high levels of acrylamide. Acrylamide was found in certain staple foods like fried potatoes and coffee as well as in baked products like potato chips, biscuits, french fries, bread, and a range of other heat-processed food products (Rosen and Hellenas 2002).

The Swedish studies revealed the ubiquitous occurrence of acrylamide in commonly consumed starch-based foods that were baked, roasted or fried leading to introduction of l-asparaginases into the food technology (Tareke et al. 2002). Acrylamide that forms during the heating of certain foods at high temperatures and low moisture conditions was considered as a potential cause of cancer (Gokmen et al. 2006). Acrylamide shows a variety of adverse effects in animals and humans. It is considered to be a neurotoxin (causing peripheral neuropathy) after occupational exposure in humans, and a reproductive toxic agent in case of rodents (Tritscher 2004). Acrylamide formation in foods is influenced by several factors that includes processing temperature, time, content and species of reducing sugars, pH, moisture content etc. (Brathen and Knutsen 2005; Pedreschi et al. 2005; Rydberg et al. 2003), indicating that acrylamide formation in foods can be controlled by reformation of the food processing technology. Therefore, producing food stuffs with low or trace levels of acrylamide without compromising with the sensorial properties of the final product seems to be the major challenge (Zhang and Zhang 2007). Generally, most of the methods used for the reduction of acrylamide content in food products mainly focus on process parameters (e.g. vacuum frying or conventional frying at low temperatures) or employing post-frying techniques which could decrease the acrylamide formation.

In potatoes, asparagine concentrations are relatively high as compared to the reducing sugar contents. Thus, it represents the limiting factor in acrylamide formation that is largely determined in potato products (Amrein et al. 2004; De Wilde et al. 2005). Therefore, for reducing the acrylamide levels in fried potatoes, one of the alternatives could be the use of LA as this enzyme hydrolyzes free asparagine into a non-precursor. In fact, treatment of raw products with LA prior to the thermal treatment resulted in a 96–99 % drop of acrylamide formation (Lindsay and Jang 2005; Zyzak et al. 2003). In recent years, the use of enzymes containing LA in potato processing has been extensively studied, so as to reduce the content of toxic acrylamides developed during the frying at high temperatures (Pedreschi et al. 2004).

LA is known to be produced by various microorganisms (Verma et al. 2007) mostly by the process of submerged fermentation and very fewer reports have suggested the use of SSF. The SSF technique has recently gained importance in production of microbial enzymes due to low energy inputs, easier downstream processing, and high volumetric productivity over conventional submerged fermentation (SmF). Moreover, solid state fermentation resembles the natural environment of microbes compare to that of synthetically designed submerged fermentation medium. Therefore, with regard to this, the present study focused on purification and characterization of l-asparaginase from *Bacillus subtilis* strain KDPS1 using SSF technology and its application in degradation of acrylamide in case of potato slices.

## Methods

### Isolation of microorganisms

Soil samples were collected from the wells near the Junagadh district, Gujarat, India. For initial enrichment, samples were further transferred to conical flask containing 100 ml of sterile seawater complex broth and were kept in the incubator shaker at 37 °C for four days. A loopful of inoculum from the pre-enriched broth was streaked on selective LA screening media (LSM) using phenol red as the indicator dye. Plates were incubated at 37 °C for 24 h. Pink color zone was observed surrounding the colonies, which was considered as the indicator of LA production.

### Bacterial identification and phylogenetic analysis

The morphological, cultural, and biochemical characteristic of the isolated strain was studied according to the Bergey’s manual of determinative bacteriology (Buchanan et al. 1974). For bacterial identification and phylogenetic analysis, genomic DNA was isolated by SDS lysozyme method (Sambrook and Russel 2001). The PCR amplification of 16S rDNA gene was performed using the forward 5′-AAGAGTTTGATCATGGCTCAG-3′and reverse primer 5′-AGGAGGTGATCCAACCGCA-3′ respectively. The amplified DNA fragment was separated on 1 % agarose gel, further eluted and purified. The amplified PCR product was sequenced and the species was identified by performing a nucleotide sequence database search using BLAST program from GenBank. Sequence data of the related species were retrieved from GenBank database. Phylogenetic tree was constructed by using the neighbor-joining method. The generated sequence was submitted in Genbank with accession number JQ964032.

### Raw material for solid-state fermentation

In the present study, soybean meal, orange peel powder, wheat straw, rice straw, sugarcane baggase, and corn cob were used as the substrates for LA production. These substrates were purchased from the nearby farmers of the Rajkot area and orange peels were collected from different fruit juice shops near Rajkot. Substrates were then dried at 60 °C overnight in a hot air oven to remove the moisture content.

#### Culture conditions and enzyme production

Production of LA was carried out by SSF. The inoculum/seed medium was prepared by adding a loopful of active culture into a 250 ml erlenmeyer flask containing 50 ml of autoclaved nutrient broth. Activated culture was inoculated in production media composed of 5 g of orange peel powder and 20 ml of 0.1 M acetate buffer (pH 5.0). The flasks were inoculated with 3 ml of the seed medium and were kept in incubator at 37 °C for 6 days. The extracellular enzyme was harvested by addition of 25 ml of 0.1 M acetate buffer (pH 5.0) followed by centrifugation at 8000 rpm for 20 min. The cell-free supernatant was used as crude enzyme preparation.

#### Effect of various physico-chemical parameters

Various process parameters like substrate concentration, type of substrates, moistening agents, and moisture ratio were optimized for maximum production of LA. Substrates were added in different quantities of 5, 7, 9, and 11 g respectively. Apart from distilled and tap water, different moistening agents such as Basal, Toyama’s, and mineral salt solutions were checked for optimizing the growth of strain on media and LA production. Also, for assessing the effect of particle size on enzyme production, various sieve sizes viz., 44, 60, 80, 100, and 120 were taken for experimentation.

### Enzyme purification

#### Ammonium sulphate precipitation (partial purification)

For partial purification, ammonium sulfate was added to the clear supernatant with constant stirring and was incubated overnight. Maximum LA activity was observed within the fraction precipitated at 60–80 % saturation. The precipitate was collected by centrifugation at 10,000 rpm for 20 min and dissolved in a minimal amount of 0.1 M acetate buffer (pH 5.0), and was dialyzed against the same buffer for 24 h. All the purification steps were carried out at 4 °C unless otherwise stated.

#### DEAE cellulose and size exclusion chromatography

The dialyzed sample was loaded onto pre-equilibrated DEAE column with 0.1 M acetate buffer (pH 5.0) for ion exchange chromatography. The adsorbed protein was eluted using a linear gradient of NaCl (0–200 mM) in 0.1 M acetate buffer (pH 5.0). The active fractions were pooled, checked for enzyme activity, and stored at −20 °C for further analysis. The protein content was determined according to the Bradford’s method (Bradford 1976). Bovine serum albumin (fraction V) was taken as standard.

#### Molecular weight determination

Electrophoresis of purified enzyme was performed as described by Laemmli (Laemmli 1970), using Bio-Rad-MINIPROTE, n-tateracell electrophoresis unit Gels with 15 × 10 cm. Protein bands were visualized by coomassie brilliant blue R-250 staining.

### Enzyme assay

LA enzyme assay was performed by a colorimetric method, according to Wriston and Yellin (1973) at 37 °C, using UV–visible spectrophotometer through estimation of ammonia produced during l-asparagine catalysis using Nessler’s reagent. Reaction mixture consisted of 0.5 ml of 0.08 mM l-asparagine, 1.0 ml of 0.1 M acetate buffer (pH 5.0), and 0.5 ml of enzyme solution. The reaction was terminated by the addition of 0.5 ml of 15 % trichloroacetic acid solution after 30 min of incubation. One LA unit (IU) was defined as the amount of enzyme, which liberates 1 µ mole of ammonia per min under the optimal assay conditions.

#### Influence of pH and temperature on LA activity and stability

The optimal pH of the purified LA enzyme was determined by measuring the activity between the pH range of 3.0–11.0. Sodium acetate buffer was used for pH 3–5, sodium phosphate buffer for pH 5.5–7, and Tris–HCl buffer for pH 8–11 was used. To test the stability of purified LA, the enzyme solution was incubated in 0.1 M acetate buffer (pH 5.0) for 10 h. Aliquots were withdrawn at every 2 h of interval. The LA activity was measured according to the standard assay method.

The optimal temperature of the purified LA was determined in 0.1 M acetate buffer (pH 5.0) and was measured at different temperature range (10–70 °C). To evaluate the stability, the enzyme solution was incubated at temperature of 10–70 °C for 10 h. Percentage relative enzyme activity was recorded at 2 h intervals during 10 h incubation.

#### Effect of metal ions and organic solvents on LA activity

In the study, the effect of various metal ions such as HgCl_2_, MnCl_2_, CuCl_2_, CoCl_2_, CaCl_2_, ZnCl_2_, MgCl_2_, NiCl_2_, and inhibitors like β-mercaptoethanol and EDTA, were studied by adding 2 mM of metal ions and 5 mM of inhibitors in the reaction mixture. The residual enzyme activities were determined after 30 min of exposure to each metal ion under the standard assay conditions. Activity was considered to be 100 % in the absence of metal ions.

### HPTLC analysis of hydrolysis product

High performance thin layer chromatography (Linomat V, Camag) was performed to study the conversion of l-asparagine into l-aspartic acid. 10 µl of samples were run in n-butanol:acetic acid:H_2_O (5:4:1) solvent system. Spots were visualized with ninhydrin reagent. Four samples: aspartic acid, asparagine, mixture (asparagine + aspartic acid) sample, and sample treated with LA, were spotted on the silica gel plate.

### Acrylamide reduction studies using purified LA enzyme

#### Experimental setup

**Fig. 1:**
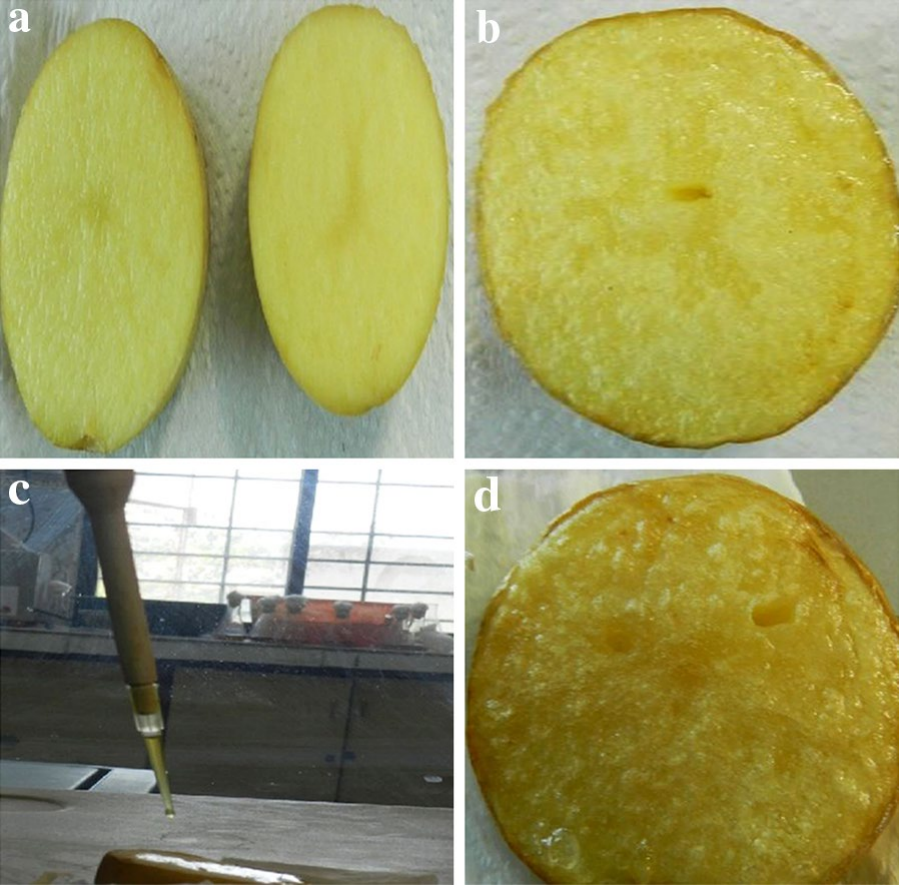
Experimental steps for performance of acrylamide degradation in potato slices. **a** Control potato slice cut (before frying). **b** Control potato slice (after frying). **c** Application of l-asparaginase on potato slices. **d** Potato slice after l-asparaginase application

The potatoes samples were cut into thick long slices and fried one at a time few millimeters above the pot bottom, without stirring, using commercial olive oil (about 100 ml) in a small steel pot (metal thickness 3 mm) on a common methane stove, at 170 °C frying temperature. The control samples without LA and the treated samples (with LA 500 μl) was fried 6 min at 170 °C (Fig. 1).

#### Formation and reduction of acrylamide determination by HPLC method

Formation and reduction of acrylamide was determined according to the method reported by Wenzal et al. (2003). 3 g of sample was processed from each group. Samples were de-fatted with hexane, then filtered through Whatmann filter paper no. 4 and were placed in a beaker with 400 μl of acrylamide internal standard (5 μg/l). After 30 min, the samples were mixed with 20 ml distilled water and placed in an ultrasonic bath at 60 °C for 15 min. Further, 20 ml of acetonitrile and 500 μl K_4_Fe(CN)_6_·3H_2_O and ZnSO_4_·7H_2_O were added to obtain a clear solution of the extract. The mixture was further centrifuged and clear supernatant was used for HPLC analysis. The 50:50 ratios of acetonitrile and 1 % formic acid were used as the mobile phase. The analysis was carried out in triplicates.

## Results and discussion

### Screening of bacterial strains for LA activity

The present study focuses on the isolation of bacterial species for production of LA enzyme with its application in degradation of acrylamide in potato chips. Fifteen different bacterial strains were isolated from 10 soil samples collected from the different wells near Junagadh district, Saurashtra region, Gujarat, India. All of the strains were initially cultured on LA specific media as reported by Prakasham et al. (2010). KDPS-I isolate (further identified as *Bacillus subtilis*) showing high growth and clear pink color zone was selected for LA production.

The isolate was aerobic, small rod, gram positive, and showed subterminal endospore on spore’s staining. The strain was mesophilic in nature and grew up to 45 °C at pH 5.0–7.0. The strain showed positive growth in medium containing glucose, arabinose, galactose, lactose, and sucrose while others showed negative results. The strain was able to hydrolyze starch and casein, utilize citrate, but could not produce hydrogen sulphide. It exhibited positive urease and catalase activity. According to the morphological and biochemical characteristics of the strain, KDPS1 was classified to be a species belonging to the *Bacillus* genus.

The phylogenetic position of the strain was determined by amplifying the 16S rDNA region and sequence of the strain was examined by BLAST analysis. The 16S rDNA gene partial sequence of the isolate was compared with the nucleotide sequences of other *Bacillus* strains retrieved from the NCBI GenBank database using the neighbour-joining method. The strain showed maximum 16S rDNA gene sequence homology (99 %) with *Bacillus* sp. A11 (NCBI Accession No. KC434967.1). Therefore on basis of the microscopic, macroscopic, biochemical, cultural, and 16S rDNA gene sequencing, the isolate was identified as *Bacillus subtilis* sp. The 16S rDNA gene partial sequence of the isolate *Bacillus subtilis* strain KDPS1 was deposited in the GenBank databases under the accession number JQ964032.

### Asparaginase production using solid state fermentation

#### Screening of different agro-industrial waste for enzyme production

**Fig. 2:**
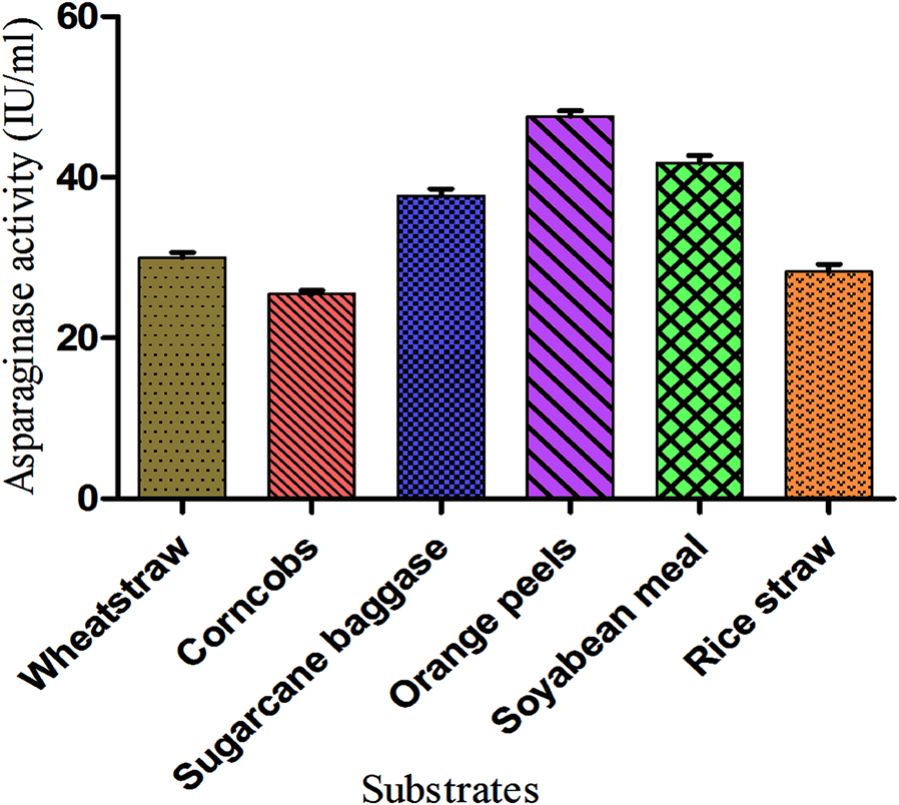
Potential of agro-industrial waste on l-asparaginase production

The selection of a suitable combination of carbonaceous (C) and nitrogenous (N) substrates is a critical factor for a SSF process (Pal and Khanum 2010). In the present study, six different substrates viz. wheat straw, orange peels, rice straw, soyabean meal, sugarcane bagasse, and corn cobs were used. Figure 2 shows the effect of all the substrates on enzyme production. Growth and production of LA was found maximum using orange peel as substrate (47 IU/ml) after 48 h of incubation at 37 °C. Presence of 20 % of soluble material apart from 20 % cellulose and 1.87 % ash content might attribute to the maximum production of LA using orange peels (Mohsen 1996). Other substrates like soyabean meal and sugarcane baggase also supported the enzyme production. For LA production, various substrates were used by different researchers depending on the need and substrate availability (El-Bessoumy et al. 2004; Hymavathi et al. 2008; Kumara et al. 2013). But apart from the choice of substrates as nutrient source for the microorganisms, characteristics like oxygen transfer and heat dispersion are also important.

#### Effect of moisture content and moistening agent on enzyme production

**Fig. 3:**
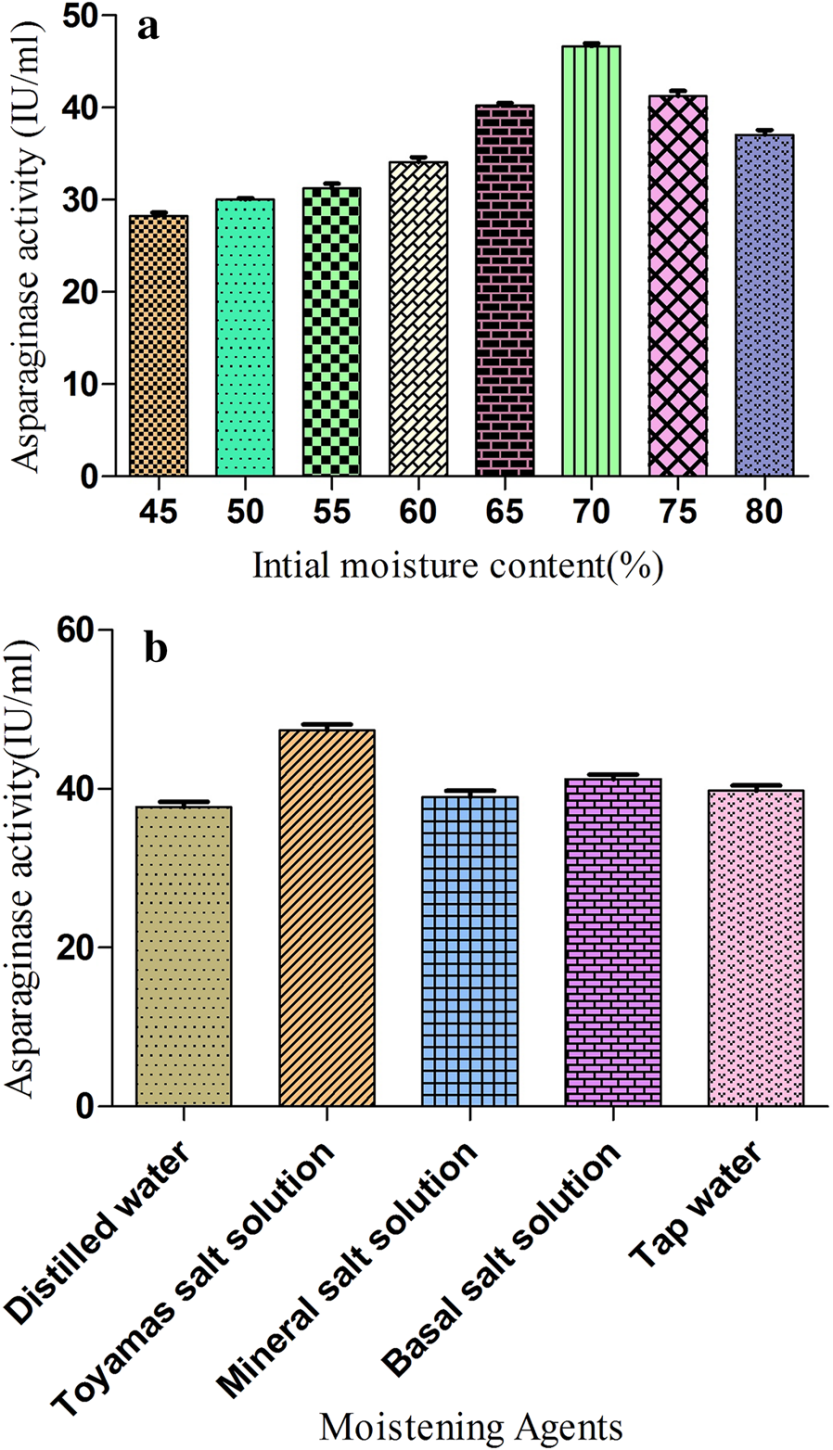
**a** Effect of different initial moisture levels on l-asparaginase production. **b** Effect of different moistening agents on l-asparaginase production

The moisture level in SSF has a great impact on the physical properties of the substrate (Pokorny et al. 1997). Many researchers had reported that the initial moisture content affects the production of hydrolytic enzymes under SSF conditions by influencing the growth of organisms (Nishio et al. 1979; Ramesh and Lonsane 1990). In the present investigation, eight moisture levels ranging from 45 to 80 % were established to study their effect on LA production and the results obtained are shown in Fig. 3a. The highest production of LA was obtained when the initial moisture content was at 70 %. Either low or high initial moisture significantly decreased the enzyme production for the reason that low moisture levels in substrates reduced the mass transfer process whereas high moisture levels in substrates reduced the porosity of the medium (Adinarayana et al. 2003). The effect of moisture content on LA production was previously studied and a requirement of 60–80 % initial moisture content for maximum LA production by *Cladosporium* sp. and *Pseudomonas aeruginosa* sp., respectively, was determined (El-Bessoumy et al. 2004; Kumara et al. 2013).

Besides distilled Water, other four moistening agents were also examined, and results (Fig. 3b) revealed that the composition of the moistening media profoundly affects LA yield. Basal salt solutions and tap water resulted in higher LA production as compared to D/W alone. Enzyme production using Toyama’s salt solution (pH 5.0) was significantly higher than other moistening agents and it was therefore selected as the moistening agent in further experiments. Our studies are in line with the reports on LA production with moistening agent (distilled water) at acidic pH using *Cladosporium* sp. (Kumara et al. 2013). Other researchers have reported LA production using buffer as moistening agent on neutral and near neutral pH range depending on the type of organism used (Soniyambi et al. 2011; Yasser et al. 2002).

#### Effect of inoculum size and incubation time for LA production

Inoculum size plays an important role in production of LA enzyme. Optimum level of enzyme production was observed at 3 % inoculum level. However, significant differences were observed when inoculum size was varied relative to that of the optimum inoculum size along with variation in the concentration. The reduction in the enzyme activity at inoculum sizes higher than optimum levels was found to be inhibitory in nature due to decrease in the concentration of medium components (Kenari et al. 2011).

As for incubation time, enzyme production started after 16 h of inoculation and showed maximum production (47 IU/ml) after 48 h of incubation at 37 °C (Table 1). The incubation time for achieving the maximal enzyme level was governed by the characteristics of the culture and was based on the growth rate of microorganisms and enzyme production (Kunamneni 2005). Short incubation time offered potential for inexpensive production of the enzyme (Sonjoy et al. 1995). With prolonged incubation, enzyme activity decreased suggesting that the end-point of fermentation should be carefully controlled because synthesized LA could be degraded by non-specific proteases secreted by the *Bacillus* sp.

**Table 1:** Effect of incubation time on l-asparaginase production (IU/ml)

Incubation time (h)	Asparaginase production (IU/ml)
12	20 ± 1.45
24	32 ± 1.09
48	47 ± 2.87
72	37 ± 1.96
96	19 ± 1.88
120	18.66 ± 0.98

#### Effect of pH and temperature on enzyme production

Determination of optimal pH and temperature is essential for the production of LA as enzymes are very sensitive to change in pH and temperature. The production of LA was found to be maximal using buffer with pH 5.0. Findings of the study are in contrast to the results reported for LA production at pH 7.95 and pH 8 by SSF using *E. aerogenes* sp. and *P. aeruginosa*, respectively (Abdel and Olama 2002; Mukherjee et al. 2000). Temperature is another important factor, which affects the enzyme production in SSF as the production process is closely related to the temperature growth optima of the microorganism. In the present study, optimum enzyme production of 47 IU/ml was observed at 37 °C which was similar to growth temperature of *B. subtilis* strain KDPS 1. Production of enzyme was found to be sustained up to 40 °C and after which a sharp decline was observed in the production of enzyme LA. The physiological changes induced by high temperatures during enzyme production are not completely understood, and previous reports suggests that at such high temperatures, microorganisms may synthesize only a reduced number of proteins essential for growth and other physiological processes (Gawande and Kamat 1999). In the present study, the temperature optima falls in the range of 25–40 °C as reported in the studies for production of LA by other researchers (Yasser et al. 2002; Dey et al. 1988; Kumar et al. 2011).

### Purification of LA enzyme

The crude filtrate obtained was subjected to ammonium sulfate precipitation. Maximal activity was observed in 80 % ammonium sulfate fractions. These fractions were dialyzed with 0.1 M acetate buffer (pH 5.0) and were subjected to further purification by ion exchange chromatography (DEAE cellulose chromatography). Purification steps are summarized in Table 2.

**Table 2:** Purification of l-asparaginase from *Bacillus subtilis* KDPS1

Steps for purification	Total protein (mg)	Total activity (U/ml)	Specific activity (U/mg)	Fold purity	% yield
Crude enzyme	5.85 ± 3.34	46.87 ± 7.12	8.01 ± 3.62	1.00	100
Ammonium sulphate precipitation	1.12 ± 2.64	41.10 ± 6.15	36.69 ± 7.44	4.58	87.61
Ion exchange chromatography	0.41 ± 1.12	39.79 ± 4.71	97.04 ± 12.28	12.11	84.89

**Fig. 4:**
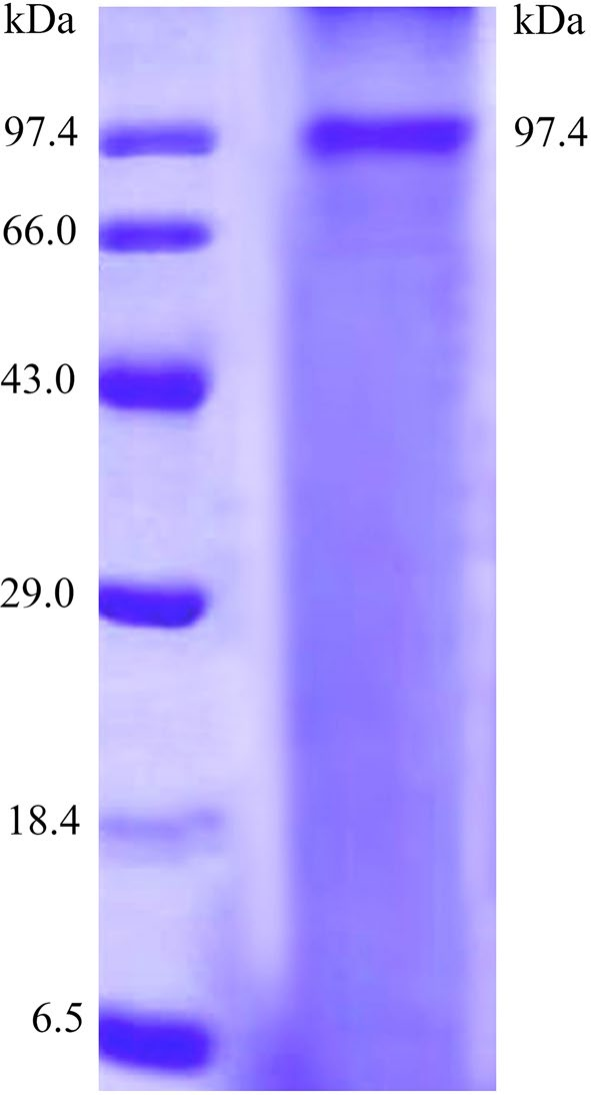
Molecular mass (97.4 KDa) determined by SDS Page

For all the purification and elution steps of LA, sodium acetate buffer pH 5.0 was used. The enzyme was purified to homogeneity after ion exchange chromatography with 84.89 % yield and 12.11 fold purity. Further, confirmation of the homogenous nature was done by SDS-PAGE, for molecular weight determination. The molecular weight of the purified enzyme was found to be 97.4 kDa. This finding was in agreement with the previous observation, in which bacterial LA existed with molecular mass in the range of 85–140 kDa (Dharmaraj and Dhevendaran 2010; Dharmaraj and Sumantha 2009). However, these results were different from the reports of LA from bacterial species having higher molecular weight range of 140–160 kDa (Kumar et al. 2011; Aghaiypour et al. 2001). The SDS-PAGE result depicted homogenous nature and medium range molecular weight of LA obtained from *B. subtilis* strain KDPS1 (Fig. 4).

#### Effect of temperature and pH on enzyme activity and stability

**Fig. 5:**
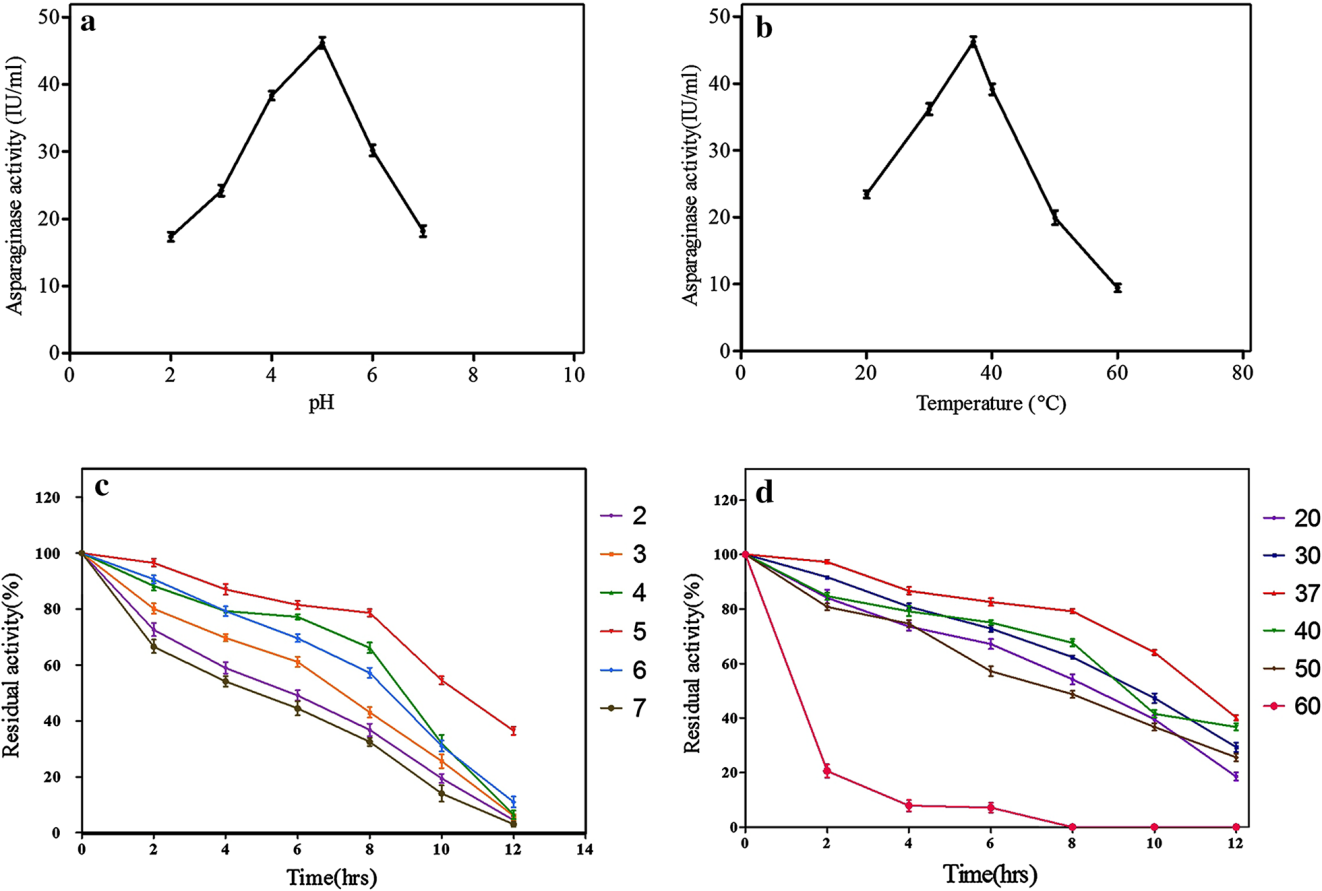
**a** Effect of pH on enzyme activity; **b** effect of pH on enzyme stability; **c** effect of temperature on enzyme activity; **d** effect of temperature on enzyme stability

Temperature and pH are the key environmental factors that most markedly influence enzyme activity. In the present study, the purified LA was found to be most active at pH 5.0 (Fig. 5) and exhibited more than 40 % of maximum activity at broad range of pH 2.0–7.0. It retained 80 % of the activity at pH 5.0 after 8 h of incubation (Fig. 5). Jung et al. (2003) showed that lowering the pH of the potato discs with citric acid before frying was an efficient way to considerably diminish the acrylamide formation in French fries. Hence considering this, purified LA (pH 5.0) can be considered as a biological substitute of organic acids for acrylamide reduction. Investigation results were in agreement with the LA enzyme extracted from *E. coli*, exhibiting an acidic pH optimum of 5.0–6.0 (Muller and Boos 1998), *Streptomyces* sp. strain PDK2 and *Streptomyces* sp. strains S3, S4, and K8 (Basha et al. 2009; Dhevagi and Poorani 2006). However, majority of previous reports suggests pH optima of LA lie in alkaline pH conditions (Kumar et al. 2011; Borkotaky and Bezbaruah 2002; Mohapatra et al. 1995).

The optimum temperature for the purified LA activity was determined by incubating the reaction mixtures at different temperatures ranging from 20 to 60 °C. The maximum activity was obtained at 37 °C (Fig. 5). Enzyme activity was found to be stable at temperature range 20–50 °C (Fig. 5). At temperatures above 50 °C no enzyme activity was retained. Inactivation at high temperatures may be due to the incorrect conformation caused by hydrolysis of the peptide chain, destruction of amino acids, or aggregation. The majority of l-asparaginases from different bacterial species showed temperature optima and stability between 30 and 50 °C (Kumar et al. 2011; Borkotaky and Bezbaruah 2002; Stepanyan and Davtyan 1998).

#### Effect of metal ions, EDTA, and inhibitors on enzyme activity

Eight different metal ions, Mg^2+^, Mn^2+^, Cu^2+^, Hg^2+^, Ca^2+^, Zn^2+^, Co^2+^, and Ni^2+^ in the form of their salts at two different concentrations of 2 mM and 5 mM, were supplemented to investigate their effects on enzyme activity. The enzyme activity was greatly inhibited in presence of Zn^+2^, CO^+2^, and Hg^+2^, and was moderately inhibited by Mg^2+^, Mn^2+^, Cu2^+^, and Ca^2+^ metal ions (Table 3). The inhibition of activity in the presence of Hg^2+^and Zn^2+^ might indicate presence of essential vicinal sulfhydryl group(s) (Kumar et al. 2011). However, the inhibition by these metal ions also indicates that enzyme is not a metalloprotein (Borkotaky and Bezbaruah 2002). The enzyme shows stimulant activity against the β-mercaptoethanol and inhibition to EDTA, which indicates the presence of cysteine residues and confirms the dependent nature of protein on metal ions. The thiol reactivity was also observed in the purified LA obtained from *Erwiniacarotovora* (Warangkar and Khobragade 2010).

**Table 3:** Effect of metal ions, inhibitors and EDTA on l-asparaginase activity

Metal ions/chemical	Relative activity (%)	
	2 mM	5 mM
Control	100	100
Ca^+2^	58	42
Hg^+2^	29	10
Co^+2^	27	13
Mg^+2^	61	57
Mn^+2^	56	49
Ni^+2^	45	36
Cu^+2^	51	39
Zn^+2^	26	09
β-Mercaptoethanol	126	110
EDTA	35	21

### Acrylamide reduction studies using purified LA

For acrylamide reduction studies, the supernatant was filtered with membrane filters (0.45 μm pore size) and the filtrate was analyzed by a HPLC embedded with ultraviolet (UV) detector. The mobile phase mixture was kept as acetonitrile and 1 % (50:50) formic acid with steady flow rate at 1 ml/min. The analysis was carried out in triplicates.

**Fig. 6:**
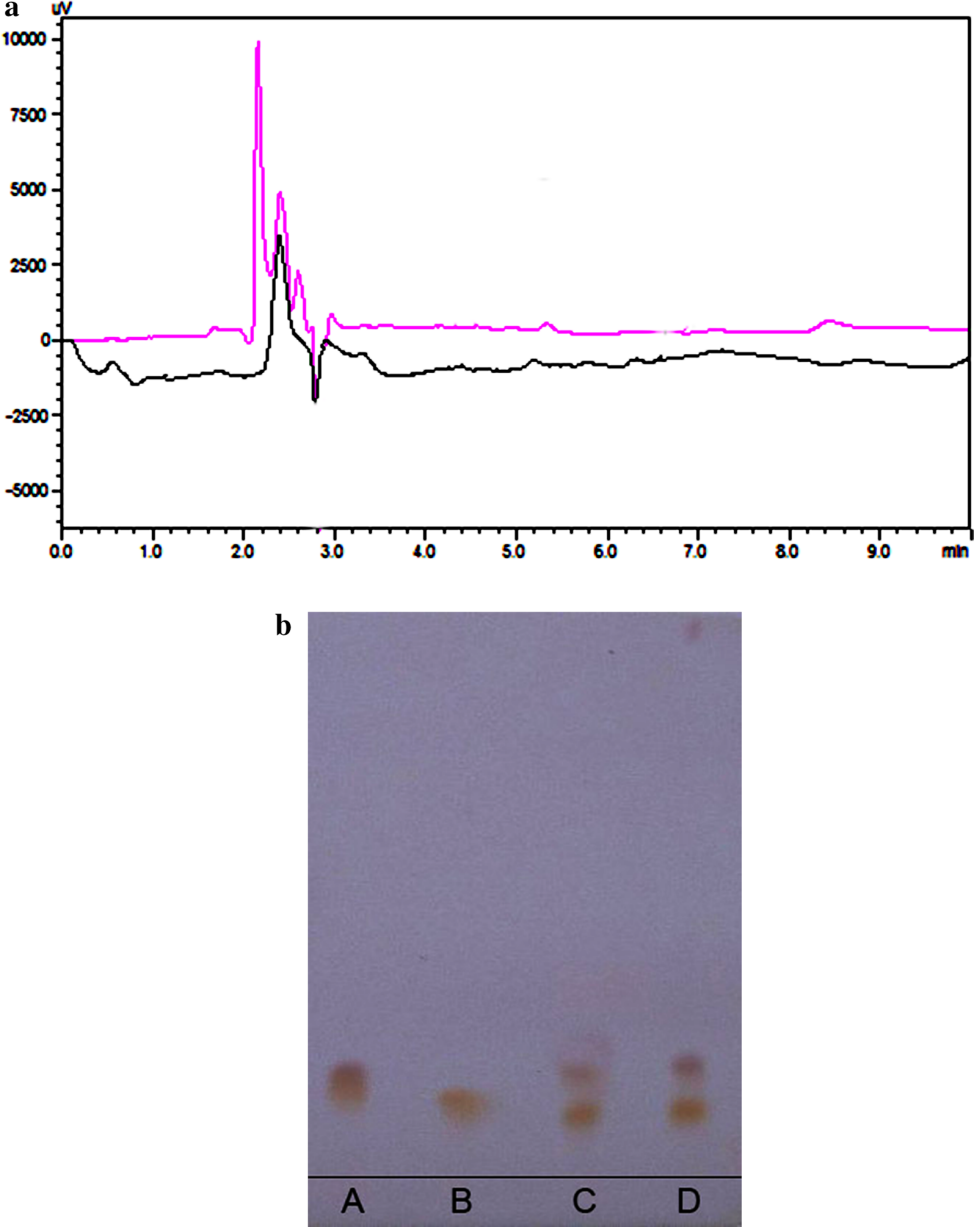
**a** It represents the chromatogram of control and sample treated with pure LA; **b** HPTLC analysis of hydrolysates of l-asparaginase enzyme. From *left*: *A* aspartic acid, *B* asparagine, *C* mixture (aspartic acid + asparagine), and *D* treated sample with LA

Figure 6a represents the chromatogram of control and sample treated with pure LA. Tables 4 and 5 shows the HPLC data discussed below. The height and area of peak no. 2 of control was recorded as 9762 mAU and 34.62 %, respectively. A compared with sample treated, height and area of Peak no. 2 was found to be 3304 mAU and 1.468 %, respectively. From the decreased values of height and area of Peak no. 2 clear indication of acrylamide reduction around 90–95 % was observed. The present investigation, uncovers the degradation of acrylamide with LA treatment leading to 90–95 % acrylamide reduction. Henceforth, on comparison to the other reported methods like blanching (Pedreschi et al. 2011) current protocol of LA usage gives more significant results in terms of acrylamide degradation.

**Table 4:** Data for control (potato slices without l-asparaginase treatment)

Sr. no.	Retention time (min)	Area (AUC)	Height (mAU)	Area %
1	1.67	8031	337	5.1381
2	2.148	54,114	9762	34.6226
3	2.395	35,061	4229	22.4327
4	2.589	8605	1540	5.5057

**Table 5:** Data for sample (Potato slices with l-asparaginase treatment)

Sr. no.	Retention time (min)	Area (AUC)	Height (mAU)	Area %
1	0.551	5652	511	2.5116
2	1.003	3304	207	1.4682
3	1.48	22,730	907	10.0997
4	1.963	220	62	0.0976

#### Analysis of hydrolysates by HPTLC

LA catalyzes the conversion of l-asparagine to l-Asp and ammonia. For identification of enzyme hydrolysates, the hydrolyzed product was spotted on HPTLC plate. The hydrolysates were characterized by ninhydrin spray reagent. For confirmation of LA enzyme, aspartic acid, asparagine, and mixture (aspartic acid and asparagine) were kept as control for the experiment. From the Fig. 6b, it was cleared that the enzyme was LA, with aspartic acid and asparagine as byproducts.

## Conclusion

Agricultural residues were transformed into value-added products by solid state fermentation using *Bacillus subtilis* sp. strain KDPS1. In the present study, LA obtained from strain KDPS1 was purified with molecular mass of 97.4 kDa. The purified enzyme showed superior activity and stability over a wide range of physiological conditions like temperature, pH, and exposure to metal ions. Acrylamide formation upon frying of potato slices treated with LA shows approximately 90–95 % drop compared to that of untreated potato slices. Further combination of LA usage with conventional process like blanching may give more beneficial results but it deserves more attention to reach commercial feasibility.
